# Xanthogranuloma of the sellar region: A case report

**DOI:** 10.1097/MD.0000000000022619

**Published:** 2020-10-02

**Authors:** Xiaotong Shao, Chao Wang, Jie Min

**Affiliations:** Department of Radiology, The Second Affiliated Hospital, Zhejiang University School of Medicine, Hangzhou, Zhejiang, China.

**Keywords:** magnetic resonance imaging, sellar region, xanthogranuloma

## Abstract

**Rationale:**

Xanthogranuloma of the sellar region is exceedingly rare, and described in only a handful of case reports. Herein, we present a case of xanthogranuloma of the sellar region to improve our knowledge for the diagnosis and management of this unusual disease.

**Patient concerns:**

A 50-year-old female presented with the symptoms of intermittent vomiting, occasional head discomfort, and diabetes insipidus of 1 month duration.

**Diagnoses:**

Magnetic resonance imaging showed a large well-defined, vase-like, heterogeneous mass in the sellar region. The lesion showed mixed signal with hierarchical signal presentation. Fluid-fluid level sign can be found within the lesion. The upper part of the lesion was hyperintense, and the lower part was hypointense on both T1-weighted images and T2-weighted images. The lesion showed no enhancement following the intravenous administration of gadolinium. The normal pituitary tissue was not clearly visible. Optic chiasm was compressed and displaced by the lesion. Initial diagnosis of pituitary macroadenoma with hemorrhage in the sellar region was made before surgery. Final diagnosis of sellar xanthogranuloma was confirmed by histopathological examination after surgical resection.

**Interventions:**

Gross total resection of the lesion was achieved using the microscope through endonasal transsphenoidal approach.

**Outcomes:**

The patient recovered well with improved binocular vision and no symptom of diabetes insipidus, and was discharged 5 days after operation.

**Lessons:**

Sellar xanthogranuloma should receive diagnostic consideration for the lesion that is a heterogeneously mixed mass with a degree of T1-weighted images hyperintense in the sellar region.

## 1 Introduction

Xanthogranuloma of the sellar region, also known as cholesterol granuloma, was appointed as a specific type of brain tumor by the World Health Organization in 2000. Histologically, xanthogranulomas characteristically consists of cholesterol crystals, foamy macrophages, giant cells, lymphocytic infiltrate, fibrous proliferation, necrotic detritus, and hemosiderin deposits.^[1]^ Xanthogranuloma of the sellar region is exceedingly rare, and described in only a handful of case reports. It has been reported that the prevalence of xanthogranuloma amongst pituitary tumor patients is at ∼0.6%.^[2]^ The rarity of this disease presents a preoperatively diagnostic challenge because of its difficulty of differentiation from other more common sellar lesions such as craniopharyngiomas and Rathke cleft cysts. Until now, preoperative radiological appearances and clinical outcomes have not been well described. Herein, we present a case of xanthogranuloma in the sellar region in a 50-year-old woman.

## 2 Case presentation

This study was approved by the ethics review board of the Second Affiliated Hospital, Zhejiang University School of Medicine. Informed written consent was obtained from the patient for publication of this case report.

A 50-year-old female presented with the symptoms of intermittent vomiting, occasional head discomfort, and diabetes insipidus of 1 month duration. On admission, she had reduced binocular vision, diabetes insipidus symptoms, electrolyte disorder with severely low sodium and potassium, decreased urine potassium, sodium and phosphorus, and increased urine calcium. Endocrine level test showed elevated cortisol by 467.2 nmol/L (normal range: 79–388 nmol/L), and decreased adrenocorticotrophin by 2.5 pg/mL (normal range: 7.9–63.3 pg/mL). Thyroid hormone test showed decreased total triiodothyronine by 0.38 nmol/L (normal range: 0.89–2.44 nmol/L), free triiodothyronine by 1.54 pmol/L (normal range: 2.63–5.70 pmol/L), total thyroxine by 57.1 nmol/L (normal range: 58.1–140.6 nmol/L), free thyroxine by 5.84 pmol/L (normal range: 9.01–19.05 pmol/L).

**Figure 1:**
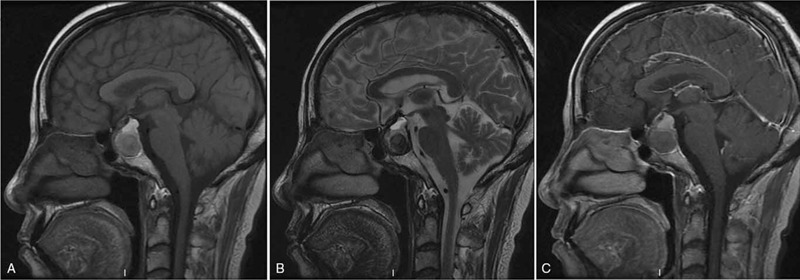
Preoperative MRI showed a large well-defined, vase-like, heterogeneous mass in the sellar region (A–C). The sign of signal stratification was found within the mass (A–C). The upper part of the mass was hyperintense, and the lower part of the mass was hypointense on both T1WI (A) and T2WI (B). The mass showed no enhancement following the intravenous administration of gadolinium (C). MRI = magnetic resonance imaging, T1WI = T1-weighted images, T2WI = T2-weighted images.

Magnetic resonance imaging (MRI) of the pituitary showed a large well-defined, vase-like, heterogeneous mass in the sellar region (Fig. 1). The sign of signal stratification was found within the mass. The upper part of the mass was hyperintense, and the lower part was hypointense on both T1-weighted images (T1WI) (Fig. 1A) and T2-weighted images (T2WI) (Fig. 1B). The lesion showed no enhancement following the intravenous administration of gadolinium (Fig. 1C). The normal pituitary tissue was not clearly visible. Optic chiasm was compressed and displaced, and the bottom of sella turcica was caved in. The diagnosis of pituitary macroadenoma with hemorrhage in the sellar region was made before surgery.

**Figure 2:**
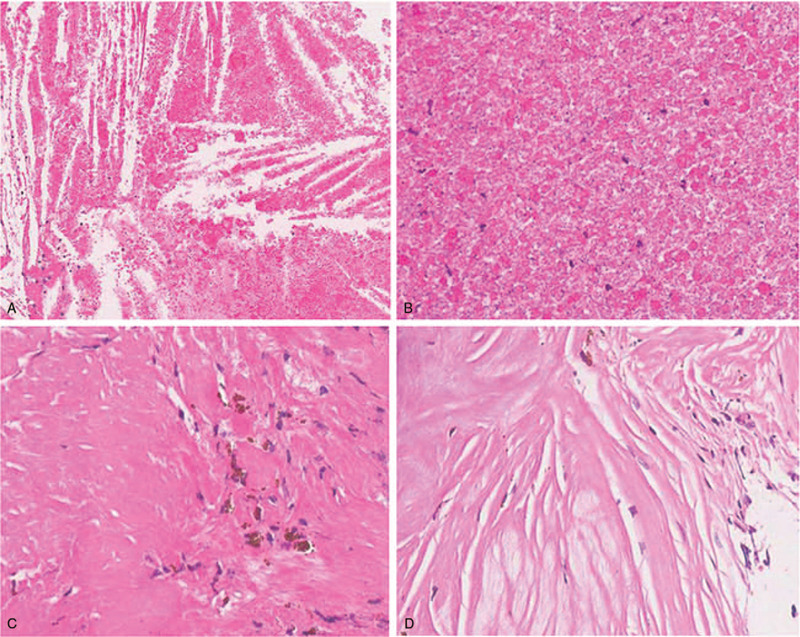
Histopathological features of the sellar xanthogranuloma. Histopathological examination showed cholesterol crystal crack (magnification, ×10) (A), necrotic tissue (magnification, ×20) (B), chronic inflammatory cells (magnification, ×20) (B), phagocytic tissue cells containing hemosiderin (magnification, ×20) (C), and collagenous fibrous tissue (magnification, ×20) (D).

Gross total resection of the lesion was achieved using the microscope through endonasal transsphenoidal approach. Histopathological examination revealed cholesterol crystal crack (Fig. 2A), necrotic tissue, chronic inflammatory cells (Fig. 2B), phagocytic tissue cells containing hemosiderin (Fig. 2C), and collagenous fibrous tissue (Fig. 2D). These pathological presentations of the lesion were compatible with the diagnosis of sellar xanthogranuloma.

**Figure 3:**
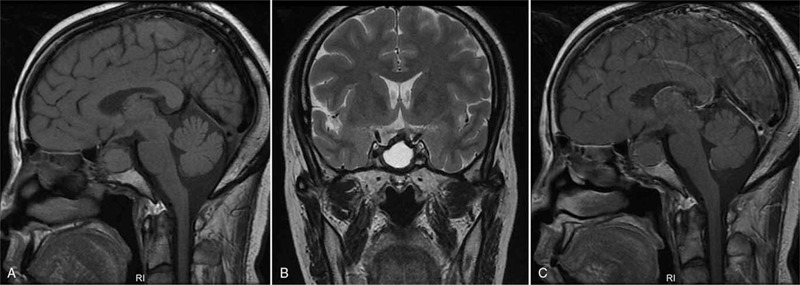
Postoperative MRI of 3 months after operation revealed complete lesion resection without residual lesion tissue (A–C). A residual cavity was formed due to postoperative fluid accumulation in the sellar region. This cavity showed hypointense on T1WI (A), hyperintense on T2WI (B), and no enhancement following the intravenous administration of gadolinium (C). MRI = magnetic resonance imaging, T1WI = T1-weighted images, T2WI = T2-weighted images.

This patient recovered well with improved binocular vision and without the symptom of diabetes insipidus, and this patient was discharged 5 days after operation. Postoperative MRI of 3 months after operation revealed complete tumor resection without residual lesion tissue (Fig. 3). A residual cavity was formed due to postoperative fluid accumulation in the sellar region. This cavity showed hypointense on T1WI (Fig. 3A), hyperintense on T2WI (Fig. 3A), and no enhancement following the intravenous administration of gadolinium (Fig. 3C). The followed-up endocrine level test showed persistent hypophysis hypothyroidism and moderately decreased adrenocorticotrophin.

## 3 Discussion

Although intracranial xanthogranulomas have been reported to occur at various sites, xanthogranuloma of the sellar region is extremely rare.^[3]^ The World Health Organization divided xanthogranuloma of the sellar region as an independent entity in 2000.^[4]^ There is controversy regarding the origin of sellar xanthogranuloma, and there are 2 main hypotheses regarding its origin: (1) It originates from an inflammatory reaction, hemorrhage, or rupture of a Rathke cleft cyst; (2) It originates from a secondary inflammatory progression of a craniopharyngioma.[^1^,^5^,^6^,^7^] The common clinical manifestations include headache, visual impairment, diabetes insipidus, vomiting, hypopituitarism, and other endocrine abnormalities. In our case, the patient had reduced binocular vision, diabetes insipidus, electrolyte disorder, and endocrine dysfunction.

There are no typical radiological characteristics or patterns for sellar xanthogranuloma.[^8^,^9^] It has been speculated that cholesterol clefts within these lesions show T1WI high and T2WI low-signal intensities[^6^,^10^]; fluid components within the cystic lesions can appear high signal on T2WI, and the development of more profound fibrosis (granulation) and hemorrhage can show low-signal on both T1WI and T2WI.^[6]^ These mixed signal intensities reflect the complex histologic components that make up granulomatous inflammatory lesions. Ved et al reported that 83% of their sellar xanthogranuloma cases showed a degree of T1W hyperintensity.^[11]^ In our case, the lesion presented as a highly heterogeneous mass with hierarchical signal presentation and fluid level sign. The upper part of the lesion was mostly hyperintense on both T1WI and T2WI which might consist of cholesterol components, and the lower part of the lesion was mostly hypointense on both T1WI and T2WI which might consist of profound fibrosis (granulation) and chronic hemorrhage. Céspedes et al reviewed the MRI findings of 33 cases of sellar xanthogranuloma, and 30 cases performed post-contrast T1WI scans.^[9]^ They found that 15 cases (50%) demonstrated no contrast enhancement; 9 cases (30%) demonstrated peripheral rim enhancement; and 6 cases (20%) demonstrated heterogeneous enhancement on the post-contrast T1WI. In our case, the lesion showed no enhancement on the post-contrast T1WI.^[9]^ Typically, Rathke cleft cyst is homogeneous on both T1WI and T2WI, and demonstrates no contrast enhancement on the post-contrast T1WI. Craniopharyngioma typically has calcification within the cystic lesion, and shows heterogeneous enhancement on the post-contrast T1WI.

Surgical resection is the current treatment for sellar xanthogranuloma, and gross total resection is considered as a gold standard treatment for this lesion.^[11]^ The endoscopic endonasal approach is generally favored over transcranial approaches.^[6]^ The visual symptoms can be effectively relieved by surgical decompression in the reported series.^[12]^ In our case, visual function was well improved 5 days after operation. However, endocrine dysfunction is relatively difficult to soon recover to the normal level.^[11]^ In our case, hypophysis hypothyroidism and decreased adrenocorticotrophin levels were persisted for 3 months during postoperative follow-up. In addition, diabetes insipidus is also a common clinical symptom of sellar xanthogranuloma, which is presumed to be a result from severe inflammatory destruction of the posterior pituitary.^[3]^ In our case, the patient had severe diabetes insipidus preoperatively, but this symptom recovered 5 days after surgical removal. Recurrence is rare after the initial operation of total or subtotal resection.[^13^,^14^] In our case, no sign of recurrence was found during 3 months of postoperative follow-up. However, as the long-term prognosis of sellar xanthogranuloma after surgical resection is yet to be evaluated, close clinical and radiological follow-up is needed.

Sellar xanthogranuloma is a rare neoplasm, and it is difficult to diagnose properly before surgery. However, sellar xanthogranuloma should receive diagnostic consideration for the lesion that is a heterogeneously mixed mass with a degree of T1WI hyperintensity in the sellar region. Surgical resection can achieve a satisfactory prognosis, effectively improve the symptoms of visual impairment and diabetes insipidus. Accumulation of more cases and long-term follow-up will be needed to further improve our knowledge for this disease.

## Author contributions

**Conceptualization:** Chao Wang.

**Data curation:** Xiaotong Shao.

**Investigation:** Xiaotong Shao.

**Writing – original draft:** Xiaotong Shao, Jie Min.

**Writing – review & editing:** Chao Wang.
